# Vertebrobasilar Dolichoectasia Presenting as Vertigo and Unilateral Weakness: A Case Report

**DOI:** 10.1155/carm/6429982

**Published:** 2026-02-15

**Authors:** Aarohi Parikh, Trishna Parikh, Ismail Hader

**Affiliations:** ^1^ Department of Internal Medicine, Healthcare Corporation of America (HCA) – Kingwood, Kingwood, Texas, USA; ^2^ Department of Internal Medicine, Case Western Reserve University/University Hospitals, Cleveland, Ohio, USA; ^3^ Department of Internal Medicine, Healthcare Corporation of America (HCA) – North Cypress, Cypress, Texas, USA

**Keywords:** case report, ischemic stroke, neurology, radiology, vertebrobasilar dolichoectasia, vertigo

## Abstract

Vertebrobasilar dolichoectasia (VBD) is a rare condition characterized by dilated and tortuous basilar and/or vertebral arteries. It can be asymptomatic or present as an ischemic stroke, hemorrhage, brainstem/cranial nerve compression, or hydrocephalus. We present a case of a 51‐year old woman with a history of transient ischemic attack who presented with persistent vertigo and left‐sided weakness and numbness for multiple days. Physical exam was remarkable for the findings of central vertigo and left‐sided impairment in sensation and strength. While computed tomography (CT) and magnetic resonance imaging were unremarkable, CT angiography showed a tortuous and dilated basilar artery to 4.9 mm, likely the underlying cause of her symptoms. She was continued on her home aspirin, clopidogrel, and high‐intensity atorvastatin and started on meclizine for symptomatic management of vertigo. Although rare, VBD should remain on the differential diagnosis for a variety of patient presentations, especially given its poor prognosis in symptomatic patients.

## 1. Introduction

Vertebrobasilar dolichoectasia (VBD) is a rare condition, whereby the vertebrobasilar arteries are dilated, elongated, and tortuous [1, 2]. VBD can be an incidental finding in asymptomatic individuals or it can present with ischemic stroke, compression of the brainstem and cranial nerves, hydrocephalus, or hemorrhage and vessel rupture [1, 2]. In one systematic review and meta‐analysis including studies of patients with nonsaccular and dolichoectatic aneurysms, the annual mortality rate was 13% per year [3]. Another study found the 5‐year case fatality risk to be 36.2%; symptomatic patients had higher 5‐year risks of case fatality, ischemic stroke, brainstem compression, and hydrocephalus compared to asymptomatic patients [4]. Therefore, patients with VBD have a poor prognosis, and treatment options are directed toward the underlying presentation. We present the case of a 51‐year old woman who presented to the hospital with the findings of central vertigo and alterations in left‐sided sensory and motor function, concerning for an ischemic etiology, though she had no evidence of an ischemic stroke on imaging.

## 2. Case

A 51‐year old woman with a history of seizures, bipolar disorder, asthma, and transient ischemic attack (TIA) currently on aspirin, clopidogrel, and high‐intensity atorvastatin presented with persistent vertigo and left‐sided weakness and numbness for multiple days. Of note, previous transthoracic echocardiogram performed the year prior showed a patent foramen ovale that had not been closed. Her vital signs were unremarkable. Physical exam was notable for bidirectional nystagmus and vertical skew on head impulse, nystagmus, and test of skew (HINTS) examination, loss of sensation in the distribution of V1, V2, and V3 on the left, a left‐sided facial droop, and diminished sensation in the left lower extremity compared to the right lower extremity. Strength was 4/5 in the left upper and lower extremities compared to 5/5 on the right side. Reflexes were 2+ in the bilateral biceps, triceps, patella, and ankles. Electrocardiogram revealed sinus bradycardia. Computed tomography (CT) of the head without contrast showed no acute intracranial abnormality. Magnetic resonance (MR) imaging of the brain was unremarkable. On CT angiography of the head and neck, the right and left vertebral arteries had no stenosis, occlusion, or dissection, while the basilar artery was tortuous and 4.9 mm in diameter with no occlusion or significant flow‐limiting stenosis (Figure 1).

Figure 1Computed tomography (CT) angiography of the head and neck in the axial (a, b) and coronal (c) planes. Arrows point to a tortuous and dilated basilar artery.

**Figure:**
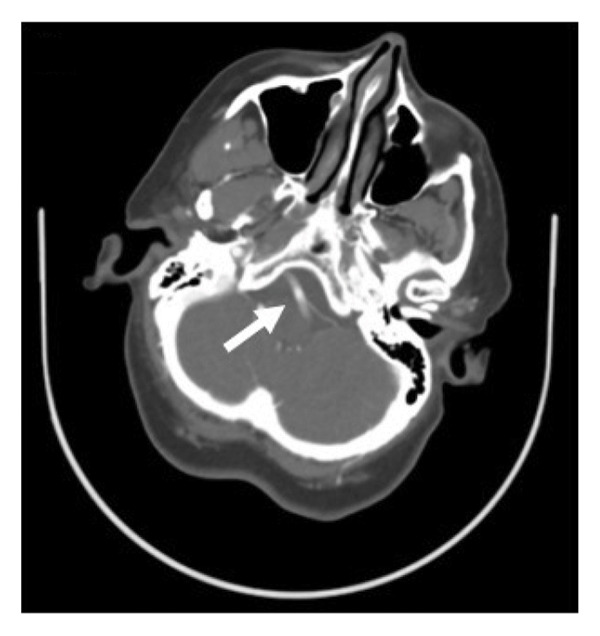
(a)

**Figure:**
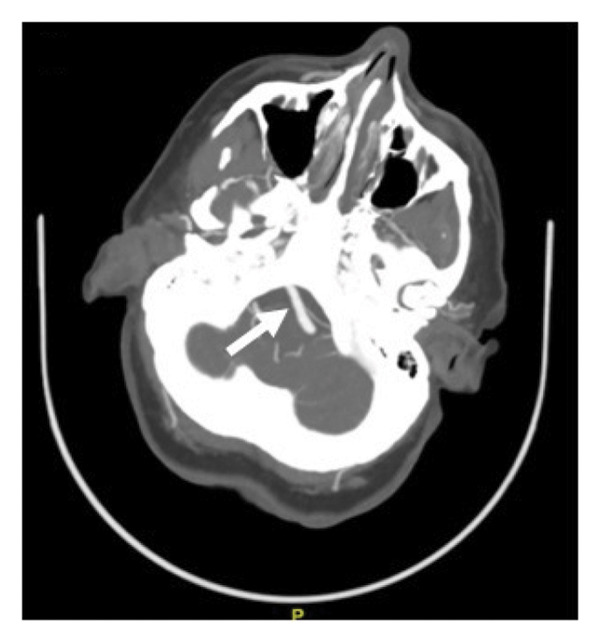
(b)

**Figure:**
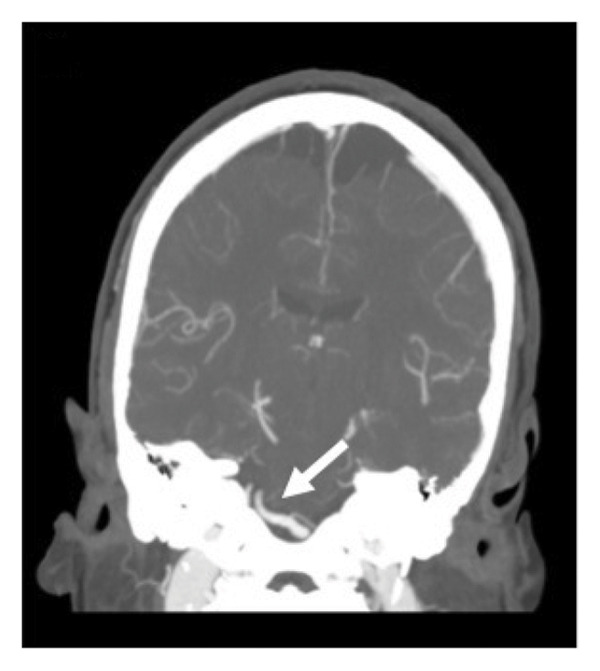
(c)

She was given 324 mg of aspirin during admission followed by continuation of her home aspirin, clopidogrel, and atorvastatin. Her vertigo was treated with meclizine. During hospitalization, physical and occupational therapy recommended inpatient rehabilitation, which she declined. She was diagnosed with symptomatic VBD and was scheduled for follow‐up with neurology outpatient. She is currently getting surveillance imaging, initially every 6 months with CT angiography of the head and neck to monitor for progression. If symptoms do not worsen, the imaging interval will be extended to yearly.

## 3. Discussion

Given the constellation of symptoms and the corresponding imaging findings, our patient likely had symptomatic VBD, presenting with ischemic symptoms. It is likely that her previous TIA was related to VBD, though this cannot be confirmed. Although it was the patient’s CT angiography that revealed a dilated, tortuous basilar artery, several imaging modalities can be used to diagnose the condition [5–7] (Table 1) with comparable results between CT/CT angiography and MR/MR angiography (MRA) [8].

**Table 1:** Radiologic criteria for computed tomography, magnetic resonance imaging, and magnetic resonance angiography.

Imaging	Description of abnormal findings
CT [5]	Basilar artery diameter at the level of the mid‐pons: ≥ 4.54 mm Basilar artery height/plane of basilar artery bifurcation: ≥ 2 grading (i.e., at least at the floor of the third ventricle above the suprasellar cistern) Basilar artery lateral positioning: ≥ 2 grading (i.e., at least lateral to lateral margin of the clivus or dorsum sellae)

MRI [6]	Basilar artery diameter: Not specified, though the mean was 6.7 ± 0.92 mm Basilar artery height/plane of basilar artery bifurcation: as per Smoker et al. [5] Basilar artery lateral positioning: Grading: 1. Midline or questionably off midline 2. Lateral displacement present 3. At the cerebellopontine angle

MRA [7]	Basilar artery: Ectasia: > 4.5 mm along the basilar artery course Basilar artery height/plane of basilar artery bifurcation: Above the suprasellar cistern Basilar artery lateral positioning: Any portion that was lateral to the margin of the clivus or dorsum sellae Basilar artery MRA findings: Abnormal if length > 29.5 mm or there was > 10 mm lateral deviation from a perpendicular line joining the start of the basilar artery to its bifurcation point Vertebral arteries: Elongated: Any portion of the vertebral artery or the basilar artery origin above the level of the pontomedullary junction, specifically if length > 23.5 mm Findings are also considered abnormal if the vertebral artery had a > 10 mm deviation from a perpendicular line connecting the start of the intracranial portion of the vertebral artery to the start of the basilar artery

Abbreviations: CT, computed tomography; MRA, magnetic resonance angiography; MRI, magnetic resonance imaging.

The pathophysiology of VBD is not well characterized; current evidence highlights genetics and infections, histological and biochemical changes, and hemodynamic factors. While VBD mostly occurs in adults, dilatation may occur in the setting of infections such as HIV and conditions such as Marfan syndrome and Ehlers–Danlos syndrome [1, 2]. Smooth muscle and reticular fiber atrophy lead to defects in the internal elastic lamina, which normally separates the intima from the media in the arterial wall [1, 2]. Other changes include a thickened intima and elastic tissue degeneration; the arterial vasa vasorum may increase [1]. This loss of elastic fibers could be secondary to an imbalance between proteases and antiproteases in the extracellular matrix [1, 2]. Wall shear stress, blood pressure, and blood flow velocity have all been suggested to play a role in the development of VBD [9]. Breakdown of the internal elastic layer, intimal thickening with neovascularization, recurrent intramural hemorrhage, thrombosis, and recanalization fuel the growth of VBD [1, 2].

Patients with VBD are often symptomatic when they are diagnosed [10]. While brainstem and peripheral nerve compression, obstructive hydrocephalus, and hemorrhage/vascular rupture can all occur, ischemic stroke is common. The 5‐year risk of TIA and stroke was 10.1% (6.3%–14.0%) and 17.6% (12.4%–22.8%), respectively, compared to subarachnoid hemorrhage (2.6%), other intracranial hemorrhage (4.7%), brainstem compression (10.3%), and progressive hydrocephalus (3.3%) [10]. VBD was associated with a 20‐fold increased odd of transient or fixed posterior circulatory dysfunction [7]. The mean diameter of the basilar artery and the height of the basilar artery bifurcation differed between those with and without a posterior circulation stroke [11]. In fact, basilar artery involvement was an independent predictor of transient or fixed posterior circulatory dysfunction [10] and recurrent stroke [12]. Alongside VBD, traditional risk factors, including hypertension and posterior circulation intracranial atherosclerosis [13], and diffuse intracranial dolichoectasia and ischemic heart disease, were risk factors for posterior circulation infarction and recurrent stroke, respectively [12].

The underlying mechanisms behind ischemic strokes in VBD are multifactorial. Slow arterial blood flow was more common in patients with VBD who had a TIA or ischemic stroke [14]. Although the differences in mean velocity, peak systolic velocity, end‐diastolic velocity, and pulsatile index did not reach statistical significance in patients with a posterior circulation stroke compared to those without a stroke, reduced blood flow alongside atherosclerosis may be underlying mechanisms for stroke [11]. Reduced blood flow predisposes to hypoperfusion and thrombus formation with subsequent clot embolization [1, 2]. Hemodynamic changes can promote atherosclerosis, while dolichoectasia alters the branching vessels, decreasing blood flow to the perforating arteries and causing cerebral infarction [2]. However, collateral circulation from unaffected portions of the basilar artery or the anterior inferior cerebellar artery, posterior inferior cerebellar artery, and superior cerebellar artery may compensate for the lack of pontine perforators at the site of the affected artery [15].

The prognosis of patients with VBD, particularly symptomatic VBD, is poor [3, 4]. VBD was associated with a 260% increased odd of all‐cause mortality (odds ratio = 3.6 [1.3–10.3]) with a statistically significant difference in Kaplan–Meier cumulative survival curves between cases and controls [7]. Treatment is often focused on treating the presenting symptoms. Our patient was continued on dual antiplatelet therapy and a high‐intensity statin. However, the risk of bleeding must be weighed with the benefits of therapy [1, 2]. For brainstem and cranial nerve compression, options include, but are not limited to, microvascular decompression, radiofrequency ablation, and botulinum toxin injection [2]. Endovascular repair is an option, though the findings suggest that it may be more beneficial in patients with hemorrhagic or ischemic symptoms rather than compression [16].

## 4. Conclusions

VBD is a rare condition characterized by dilated, elongated, and tortuous arteries of the vertebrobasilar system. Presenting symptoms are varied and include no symptoms, TIA or ischemic stroke, brainstem or cranial nerve compression, and hemorrhage. It can be diagnosed through multiple different imaging modalities. The prognosis is overall poor, particularly in symptomatic patients, and treatment is focused on the underlying symptoms. VBD should remain on the differential diagnosis in patients with any of these clinical presentations, and management strategies should be tailored to optimize outcomes.

## Funding

No funding was received for this manuscript.

## Conflicts of Interest

The authors declare no conflicts of interest.

## Data Availability

The data that support the findings of this study are available from the corresponding author upon reasonable request.
